# The Role of a Simulation-Based Activity on Student Perceptions of Parenteral Nutrition Education

**DOI:** 10.3390/pharmacy8030123

**Published:** 2020-07-21

**Authors:** Genene Salman, Henry Hua, Michelle Nguyen, Sandy Rios, Elvin A. Hernandez

**Affiliations:** College of Pharmacy, Marshall B. Ketchum University, Fullerton, CA 92831, USA; hhua@ketchum.edu (H.H.); michellenguyen.2020@ketchum.edu (M.N.); sandyrios.2020@ketchum.edu (S.R.); ehernandez@ketchum.edu (E.A.H.)

**Keywords:** simulation, parenteral nutrition, pharmacy education, active learning

## Abstract

**Introduction**: Parenteral nutrition (PN) education in pharmacy schools and postgraduate programs may not sufficiently prepare future pharmacists for clinical practice. Limited data exist regarding innovative teaching strategies in the area of PN. The purpose of this study was to identify students’ perceptions of a simulated PN activity in a pharmacotherapeutics course. **Methods**: Second-year Doctor of Pharmacy (PharmD) students from two cohorts (N = 84 for both cohorts) completed a PN assignment using simulated PN materials, which resembled those seen in clinical practice. Before and after the activity, students completed identical surveys about their perceived competence and interest in PN, which were analyzed using Wilcoxon signed-rank tests. **Results**: Following the simulation, the percentage of students affirming their perceived competence (selecting strongly agree or agree in the survey) in their ability to describe the process of combining ingredients to make a PN admixture (45.2% vs. 83.3%, *p* < 0.001) and calculate PN-related problems (58.3% vs. 83.3%, *p* < 0.001) improved. The proportion of students expressing interest in PN increased after the simulation (78.6% vs. 86.9%, *p* < 0.001). **Conclusion**: A simulated practicum experience in PN was viewed positively by PharmD students at this university, and may be a valuable active learning experience to incorporate in a PharmD curriculum.

## 1. Introduction

Nutrition support therapy refers to the use of enteral (through a feeding tube) or parenteral nutrition (PN) when patients cannot receive adequate nutrition orally for the purpose of treating or preventing malnutrition [1]. A subset of nutrition support, PN is the delivery of nutrients intravenously and consists of macronutrients (dextrose, amino acids, and injectable lipid emulsion) and micronutrients (electrolytes, vitamins, and trace elements). PN may also contain additives (e.g., insulin, famotidine). PN is reserved for patients who are unable to obtain adequate nutrition enterally or orally, and may be given to patients in an institutional setting or at home. Parenteral nutrition includes both peripheral PN (PPN) and central PN (CPN). CPN has also been referred to as total parenteral nutrition (TPN). CPN is typically more concentrated than PPN, and should be infused through a central catheter. Not only are the individual components of PN itself associated with complications, but it is also a complex medication because many ingredients and steps are involved in its preparation [2]. The Institute of Safe Medication Practices (ISMP) considers PN preparations high-alert medications due to their potential to cause significant harm when used in error [2,3].

Pharmacists working in the hospital are likely to encounter PN [4,5]. In a survey conducted by the American Society of Health-System Pharmacists (ASHP), approximately 86.3% of hospital pharmacy directors in the United States reported that PN was compounded either internally or externally (through an outsourcing facility), while the remainder of the respondents (13.6%) stated that their institutions did not compound PN [4]. Additionally, according to a recent survey by ASHP, 48.6% of hospital pharmacy directors in the United States indicated that pharmacists managed nutrition support therapy at their respective institutions [5]. 

The literature suggests that pharmacists may not be adequately prepared to provide direct patient care in the area of nutrition support [6,7]. For example, in a study by Mutz et al., pharmacy residency directors were asked to evaluate their postgraduate year one (PGY1) residents’ clinical knowledge [6]. The average score for nutrition support was 3.05 out of a maximum of 5, which was lower than their biostatistics and pharmacokinetics scores [6]. In another study, only 39.5% of the pharmacists at the beginning of a continuing education course indicated that they felt comfortable calculating protein and caloric requirements for patients on PN [7].

The American College of Clinical Pharmacy (ACCP) classifies content areas such as deficiencies and excessive intake of nutrients as tier one topics, indicating that instruction in PharmD programs should be at a depth that prepares future pharmacists to provide direct patient care [8]. ACCP lists malnutrition prevention and treatment strategies such as PN as a tier two topic, which may necessitate postgraduate training (or equivalent experience) to produce practice-ready pharmacists [8]. However, real-world learning opportunities in PN may not be offered in postgraduate programs [9]. Currently, postgraduate year two (PGY2) residency positions in nutrition support in the United States no longer exist [10]. Patient care learning experiences in nutrition support are required in PGY2 residencies such as critical care and pharmacotherapy, but PGY1 programs may not offer these opportunities to their pharmacy residents [9]. PN training is not mandatory in introductory and advanced pharmacy practice experiences (IPPEs and APPEs, respectively) in Doctor of Pharmacy (PharmD) programs [11]. Therefore, students may only be exposed to PN during the didactic portion of pharmacy school. It is crucial that educational institutions provide students with a foundation that engages and motivates them to develop a deep understanding of topics as they progress through their careers [11]. 

PharmD programs should emphasize patient-specific calculations relating to nutrients, which is required by the Accreditation Council for Pharmacy Education (ACPE) [11]. The North American Pharmacy Licensure Examination (NAPLEX) specifically tests students’ ability to perform such calculations, as part of Objective 2.1.1. [12]. The NAPLEX also examines students’ mastery of other aspects of PN (a sterile compound), such as calculations involving drug concentrations, quantities of drug required to make the final product, and rates of administration [12]. 

Moreover, the NAPLEX tests on students’ knowledge of compatibility and stability characteristics of sterile products, which may include PN [12]. Sterile compounding courses have incorporated the preparation of PN [13,14]. However, sterile compounding courses require extensive planning, time, physical space, supplies, and trained personnel. To the authors’ knowledge, there are no studies investigating the use of simulated materials alongside didactic education of PN in a pharmacotherapeutics module.

Simulation is a type of active learning strategy that engages students by immersing them in an environment that emulates real-life experiences [15,16]. Simulations have been shown to enhance knowledge retention, clinical skills, and interprofessional collaboration using a variety of tools such as high-fidelity mannequins, standardized patients, and replica models [17,18,19,20,21]. In recent years, PharmD curricula have increased their usage of simulations in a variety of subject areas [15,16,17,18,19,20,21]. Moreover, ACPE requires PharmD programs to prepare students for real-world practice and provide active learning experiences as an integral component of the didactic education [11].

In the current effort, the primary author implemented a PN simulated activity for second-year PharmD (P2) students. This activity consisted of realistic props such as simulated intravenous bags and vials commonly seen in clinical practice to supplement calculation-based PN problem sets. The purpose of this report was to demonstrate the value of a simulation-based activity in PN education, as perceived by the students.

## 2. Materials and Methods 

### 2.1. Participants

The PN education simulated activity was administered as part of a required course, “Integrated Pharmacotherapeutics III” offered to P2 students. For the current report, we present the data from two consecutive cohorts. The graduating class of 2020, “Cohort 1”, consisted of forty students, and they completed the activity during Winter 2017. The next group, “Cohort 2”, represented the class of 2021, consisting of fifty students that were enrolled in the class during Winter 2018. The Integrated Pharmacotherapeutics III course included didactic material pertaining to foundational concepts in nutrition support, particularly PN, such as the diagnosis of malnutrition, nutritional assessment and screening, PN-specific macronutrients, PN-specific micronutrients, PN administration, calculation-based problem sets, and PN complications. Cohort 1 and Cohort 2 received five hours and six hours, respectively, of didactic PN education. Additionally, all students successfully completed a compounding course in the previous academic year, which included hands-on training in aseptic technique and sterile compounding. We planned on analyzing both cohorts as one sample if the demographic data showed no statistically significant difference in baseline characteristics between the two groups.

### 2.2. Methods

This simulated activity incorporates several domains from the Center for the Advancement of Pharmacy Education (CAPE) 2013 standards: 1.1 (Learner), 2.2 (Medication use systems), 3.1 (Problem Solving), 3.6 (Communication), and 4.1 (Self-awareness) [22]. The objectives of the simulation were as follows:

1. Using the simulated materials, describe the process of combining individual PN ingredients together to form PN admixtures;
2. Given a PN order and stock solution concentrations, calculate relevant PN parameters such as the required volume of macronutrients and micronutrients (given the amount in the order), calories and percentage calories for each of the macronutrients, and percentage weight-in-volume (*w/v*) of each macronutrient in the final PN admixture preparation;
3. Demonstrate the order in which calcium gluconate and phosphate salt are added to PN to prevent calcium and phosphorus precipitation;
4. Create a positive and engaging experience that stimulates interest in PN.

Immediately after the completion of the didactic portion of PN instruction and just prior to initiating the simulated activity, students were asked to complete a pre-intervention survey. The survey consisted of five statements, which were created by the authors. Students rated their endorsement of statements about their own perceived competence and interest in PN. The survey items were:

1. “I can explain the sterile compounding and aseptic technique procedures involved in the preparation of PN”;
2. “I can explain the process in which different components (dextrose, amino acids, injectable lipid emulsion, electrolytes, vitamins, and trace elements) are combined together in the preparation of PN”;
3. “I can explain the role of the pharmacy personnel (pharmacy technician, intern, pharmacist, etc.) in the preparation of PN”;
4. “I am comfortable performing the calculations of the individual components of PN”;
5. “An elective in PN would be a beneficial course to have in the College of Pharmacy curriculum”.

Participants responded to each statement on a five-point Likert scale, ranging from 1 (Strongly Disagree) to 5 (Strongly Agree). The survey had no impact on the students’ grades. After the students completed the pre-intervention survey, students were divided into groups composed of approximately four to six students of their choice. Three to four proctors were available during the activity to answer any questions. The proctors were provided an overview of the assignment prior to the activity. 

**Figure 1:**
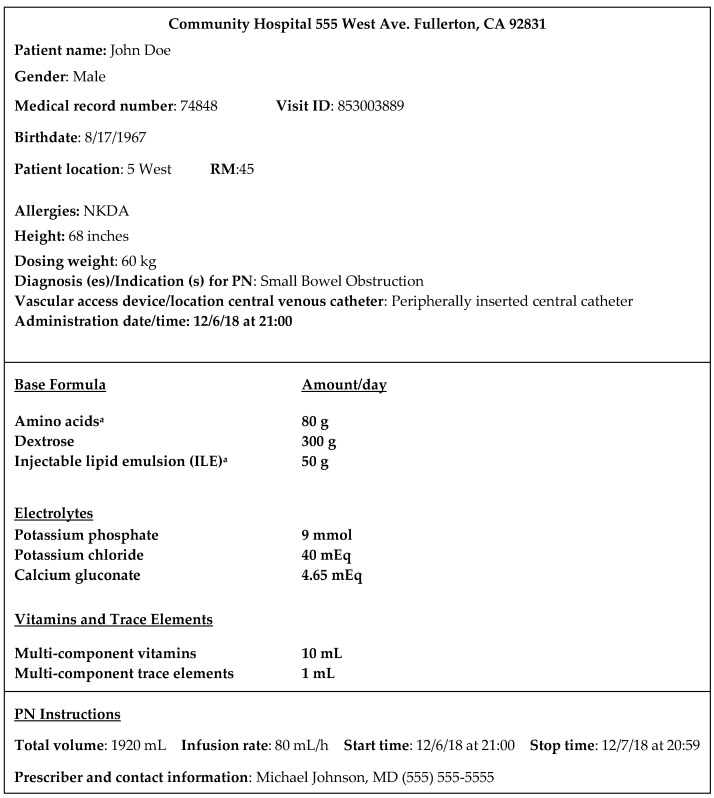
Example of the PN order used for the simulated assignment. ^a^ According to ASPEN, the actual product name should be specified [23]. **Source**: Adapted with permission from Ayers P, Adams S, Boullata J, et al. ASPEN parenteral nutrition safety consensus recommendations. *JPEN.* *J. Parenter. Enteral. Nutr*. **2014**, *38*, 296–333.

**Figure 2:**
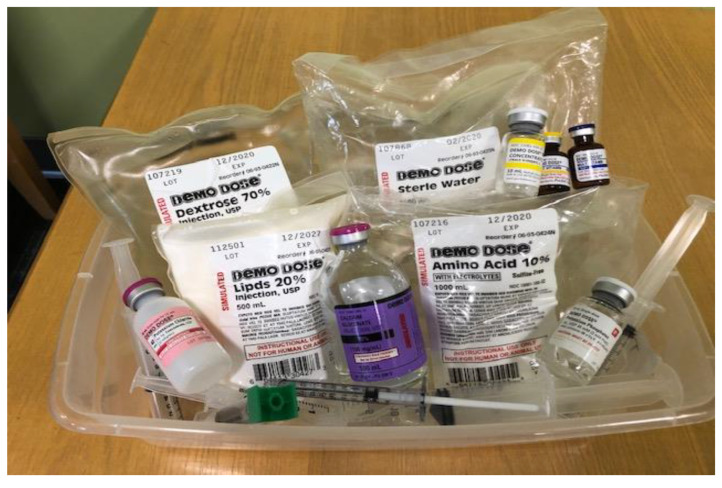
In-class, simulated supplies containing replica PN ingredients and syringes.

Students were given paper-based PPN and CPN orders, which were adapted from the American Society for Parenteral and Enteral Nutrition’s (ASPEN) order template (Figure 1) [2]. The orders listed the type of PN ingredient with the respective amounts, rate of infusion, and patient-specific data, as would be seen in clinical practice (Figure 1). Students were provided a list of the available stock solutions with the respective concentrations. Along with the list, student groups were supplied a bin containing the stock solutions from the Demo Dose^®^ TPN Compounding Kit (Pocket Nurse, Monaca, Pennsylvania, PA, USA) and syringes corresponding to the PN orders (Figure 2). The Demo Dose^®^ TPN Compounding Kit (Pocket Nurse, Monaca, Pennsylvania, PA, USA) included intravenous electrolyte vials and intravenous bags of injectable lipid emulsion, concentrated dextrose and amino acid solutions, and intravenous sterile water. These replica materials resemble the supplies commonly seen in practice. For Cohort 1, four CPN and PPN bins each were created to fulfill the orders. Since Cohort 2 was a larger class than Cohort 1, the simulated activity used five CPN and PPN bins each.

**Figure 3:**
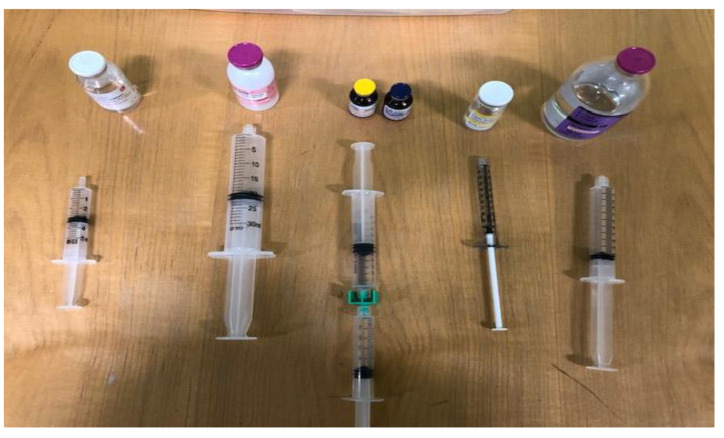
Manual set-up of micronutrients during PN-simulated activity.

The main assignment consisted of calculations of various PN parameters such as required volume of macronutrients and micronutrients (given the amount in the order), calories and percentage calories for each of the macronutrients, and percentage weight-in-volume (*w/v*) of each macronutrient in the final PN admixture preparation. Mimicking the manual preparation of PN, students were required to demonstrate the proper manual PN set-up to a proctor (Figure 3). They were required to measure accurate volumes of the micronutrients with the use of syringes and to position the phosphorus salt vial first and calcium gluconate vial last to avoid precipitation. It is important to note that ASPEN recommends injection of intravenous multivitamins just prior to administration of the PN admixture because of its short stability [23]. However, for simplicity, we assumed that the PN bag would be administered immediately after preparation. Using intravenous tubing sets, students were provided the opportunity to make a 2-in-1 (dextrose and amino acids) or 3-in-1 (dextrose, amino acids, and injectable lipid emulsion) PN admixtures using the gravity method with faculty assistance. Furthermore, using an intravenous tubing set, a nurse practitioner explained the procedure for the infusion of PN. The entire activity lasted approximately two hours. Additional PN calculation-based problem sets were provided for further practice. Students were then asked to complete the post-intervention survey, which was identical to the pre-intervention survey. A unique and anonymous identifier number linked each student’s pre-intervention survey and post-intervention survey. Students also completed a demographic survey, which helped to identify student learning preferences and background. All surveys for this study were administered on paper and were anonymous. At the end of the class, the correct answers were posted, and students were asked to self-grade their assignments. 

The average cost of PN supplies was approximately $11.50 per student for Cohort 1. Since students did not inject the micronutrients directly into the bags, those materials were reused for the following year. However, Cohort 1 combined the replica macronutrients to make the 2-in-1 or 3-in-1 PN admixtures, and those materials needed to be replaced. To carry out the simulation for Cohort 2, approximately $340 was used to replace the materials from Cohort 1 and to account for the larger class size.

### 2.3. Data Analysis

All data analyses were performed on SPSS^®^ Statistical Software Version 26 (IBM^®^, Armonk, New York, NY, USA). All statistical tests utilized a two-tailed level of significance of alpha = 0.05. Unpaired *t* tests compared exam performance between Cohort 1 and Cohort 2, Chi-square tests compared demographic characteristics between the cohorts, and Wilcoxon signed-rank tests compared the students’ survey responses before and after the simulated activity. Although the Class of 2021 utilized the anonymous identifier number to link the demographic survey with the pre- and post-intervention surveys, this was not implemented for the Class of 2020. Therefore, we did not link demographic data to survey data for Cohort 1. For consistency, demographic information and survey data were analyzed separately. The study protocol was approved by our University’s Institutional Board Review for human protections. 

## 3. Results

There were forty and fifty students enrolled in Cohort 1 and Cohort 2, respectively. Some students did not submit completed surveys or were absent from class. Four students in Cohort 1 and two students in Cohort 2 were not present or did not complete the demographic survey. The demographical information for Cohort 1 (n = 36) and Cohort 2 (n = 48) are listed in Table 1. The total sample with both cohorts is N = 84. Thirty-three (91.7%) students and forty-five (93.8%) students in Cohorts 1 and 2, respectively, obtained a bachelor’s degree. Five (13.9%) students and ten (20.8%) students in Cohorts 1 and 2, respectively, reported working as a pharmacy intern. Twelve (33.3%) students in Cohort 1 and twenty-four (50%) students stated that they had more than two learning preferences. Chi-square analyses on all demographic characteristics found no statistically significant differences between the two cohorts. Four students in Cohort 1 and two students in Cohort 2 did not submit the pre- and post-intervention surveys, resulting in exclusion from the statistical analysis.

**Figure 4:**
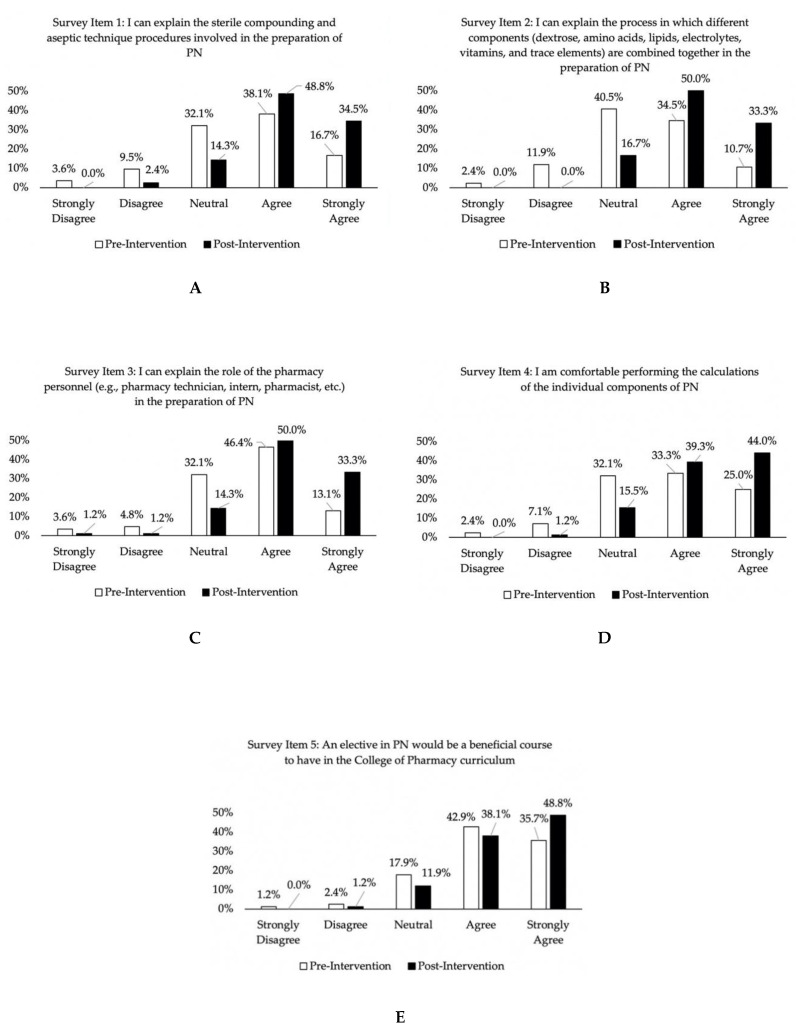
Survey responses comparing pre-intervention to post-intervention perceptions of PN, *N* = 84^a^. (**A**) Survey item 1 results; (**B**) Survey item 2 results; (**C**) Survey item 3 results; (**D**) Survey item 4 results; (**E**) Survey item 5 results. ^a^ Data were combined for Cohort 1 (*n* = 36) and Cohort 2 (*n* = 48).

All five items of the student perception surveys for the combined cohorts showed improvement (i.e., higher scores) when comparing the post-intervention survey with pre-intervention ratings. Wilcoxon signed-rank test *z*-scores for Survey Items 1–4 and 5 were −5.48, −6.19, −5.29, −5.36, and −3.43 respectively; all *p*-values < 0.001 (see Figure 4A–E for the percentage of endorsement for each of the survey items).

Students provided written feedback about the simulation anonymously on the perceptual surveys, course evaluation, or faculty evaluation. Since all surveys and evaluations were anonymous, it was possible that the same student responded more than once. The comments were generally positive, and are presented in Table 2 with minor corrections for grammar and spelling. 

## 4. Discussion

To our knowledge, this is the first study investigating the incorporation of a simulation specific to parenteral nutrition in a PharmD pharmacotherapeutics course. Similar to other studies that used simulation to enhance traditional didactic lectures [17,19], our results showed that the students were receptive to this learning strategy. Our study indicated that the majority of students believed that the PN simulation increased their confidence in PN-related tasks (Figure 4A–E). Moreover, since the simulation was a group activity, students had the opportunity to learn from and with each other. 

With the relative shortage of learning opportunities in PN in postgraduate training and the deficiencies in knowledge in PN, it is important to maximize the didactic education of PN during pharmacy school. Although it is unclear if the teaching methodology is contributing to the inadequacy of PN education, leaders in nutrition support have encouraged educators to use innovative teaching methodologies to deliver PN instruction [24]. Further studies are needed to determine if innovative strategies in PN education impact pharmacists’ competency in PN. However, following ACPE’s standards to utilize active learning strategies to engage students, the simulated activity described in this article is an option [11].

Although the simulation showed encouraging results, there were limitations. This study was conducted at a small pharmacy school, with each cohort only having one section. Therefore, the study lacked a control group of students who did not complete the simulation. Moreover, there were no pre- and post-intervention assessments to evaluate the impact on the simulation on learning outcomes. Future studies may shed light on pre-intervention to post-intervention improvement in learning outcomes with the administration of assessments. 

Although the students expressed satisfaction with this learning strategy, such an activity requires extensive time, trained personnel, and cost, all of which may contribute to the difficulty in carrying out this activity. Compounding PN admixtures in a cleanroom-like setting provides an opportunity that more accurately reflects the manner in which PN is prepared in clinical practice. Due to limitations in time, physical space, trained personnel, and financial resources, compounding PN admixtures in a cleanroom-like environment was not possible. 

With the relative scarcity of learning opportunities in nutrition support, particularly PN, in postgraduate training, we sought to create an experience that heightens students’ interest in this topic. To further prepare students for clinical practice, the incorporation of PN topics such as complex nutritional assessments and PN order review will be considered in the future. An elective course covering advanced topics in PN is also being considered. Additionally, our institution offers APPE rotations in which pharmacists are involved in the management and preparation of PN. Due to the positive response of this activity, we believe it would be appropriate for this institution and others to incorporate a simulated PN activity in a PharmD curriculum.

## 5. Conclusions

The simulated learning activity using PN materials was generally positive for students. It may be beneficial to incorporate simulation-based learning activities as a supplement to the didactic instruction of PN. Further studies are needed to confirm these results.

## Figures and Tables

**Table 1:** Demographic data for Cohort 1 (*n* = 36) ^a^ and Cohort 2 (*n* = 48) ^b^.

Variable	Cohort 1 n (%)	Cohort 2 n (%)	Chi-Square *p-*Value
Gender			0.74
Male	15 (41.7)	17 (35.4)	
Female	18 (50)	28 (58.3)	
Prefer not to disclose	3 (8.3)	3 (6.3)	
Age			0.56
20–24	4 (11.1)	9 (18.8)	
25–34	25 (69.4)	34 (70.8)	
35–44	2 (5.6)	1 (2.1)	
Prefer not to disclose	5 (13.9)	4 (8.3)	
Bachelor’s degree			0.92
Yes	33 (91.7)	45 (93.8)	
No	2 (5.6)	3 (6.3)	
Information not disclosed	1 (2.8)	0 (0)	
Bachelor’s degree subject area			0.07
Basic sciences	17 (47.2)	34 (70.1)	
Humanities or social sciences	5 (13.9)	4 (8.3)	
Other	8 (22.2)	7 (14.6)	
Information not disclosed	4 (11.1)	0 (0)	
Not applicable	2 (5.6)	3 (6.3)	
Work experience—pharmacy technician			0.65
Yes	2 (5.6)	4 (8.3)	
No	33 (91.7)	44 (91.7)	
Information not disclosed	1 (2.8)	0 (0)	
Work experience—pharmacy intern			0.44
Yes	5 (13.9)	10 (20.8)	
No	30 (83.3)	38 (79.2)	
Information not disclosed	1 (2.8)	0 (0)	
Ideal way of learning			0.10
Watching a video	1 (2.8)	1 (2.1)	
Hands-on training	6 (16.7)	10 (20.8)	
Listening to a lecture	9 (25)	3 (6.3)	
Watching a video and hands-on training	4 (11.1)	9 (18.8)	
Watching a video and listening to a lecture	2 (5.6)	1 (2.1)	
Hands-on training and listening to a lecture	2 (5.6)	0 (0)	
Watching a video, hands-on training, and listening to a lecture	12 (33.3)	24 (50)	

^a^ Four students did not complete the survey in Cohort 1. ^b^ Two students did not complete the survey in Cohort 2.

**Table 2:** Feedback from students ^a^.

Comments
I really enjoyed that the instructor brought in the PN bags.
The instructor made learning exciting and engaging by bringing in mock medications for us to practice.
PN lab was informative.
We were taught how to fill PN, but we did not have practice in writing them. It would have been a good opportunity for a cumulative case study.
I liked the visual education with PN.
This hands-on activity enhanced what we learned about PN. It was helpful to do the calculations, draw out the volume, and set it up as if we were in the hospital.

^a^ Comments were edited for grammatical or spelling errors.
